# The Associations of Long-Term Temperature and Precipitation with Chronic Respiratory Symptoms: Projections for the Changing Climate

**DOI:** 10.1007/s00408-024-00763-6

**Published:** 2024-11-29

**Authors:** Heikki O. Koskela, Johanna T. Kaulamo, Anne M. Lätti

**Affiliations:** 1 School of Medicine, University of Eastern Finland, POB 1627, 70211 Kuopio, Finland; 2 Unit for Medicine and Clinical Research, Pulmonary Division, Kuopio University Hospital, PL 100, 70029 Kuopio, KYS Finland

**Keywords:** Cough, Chronic cough, Asthma, Wheezing, Rhinitis, Chronic rhinosinusitis, Sleep apnea, Climate, Climate change

## Abstract

**Purpose:**

To clarify the associations of climatic indices with chronic respiratory symptoms, with a final aim to approximate the effects of climate change on them.

**Methods:**

An e-mail survey was directed to the members of the Finnish Pensioners` Federation. The mean 20-years’ precipitation and temperature in each subjects’ home municipality were obtained from the Finnish Meteorological Institute, separately for summer and winter. Adjusted multivariate models were utilized to investigate the associations of the climatic indices with chronic rhinosinusitis, chronic cough, wheezing with dyspnea, and sleep apnea.

**Results:**

There were 6189 responders from 283 municipalities. Chronic rhinosinusitis and chronic cough were most prevalent in the southeastern regions of the country, where the precipitation counts were highest. In the multivariate models, winter precipitation in the home municipality increased the risks of chronic rhinosinusitis and chronic cough [adjusted OR 1.80 (1.30–2.51) per 100 mm, *p* < 0.001, and 1.57 (1.19–2.07) per 100 mm, *p* = 0.001, respectively]. Wheezing with dyspnea and sleep apnea were not associated with the climatic indices.

**Conclusion:**

Chronic rhinosinusitis and chronic cough were associated with long-term winter precipitation. Given the anticipated increase in winter precipitation in Northern America and Northern Europe, the prevalences of chronic rhinosinusitis and chronic cough may increase there.

**Supplementary Information:**

The online version contains supplementary material available at 10.1007/s00408-024-00763-6.

## Introduction

The temperatures are rising globally, accompanied by changes in precipitation patterns [1, 2]. The speed of global warming is especially rapid in the arctic area, including Northern America and Northern Europe. This is accompanied with an increase in precipitation, especially during the wintertime [3–5]. Respiratory symptoms constitute a large proportion of reasons why people seek medical attention [6]. Therefore, any effect of the climate change on their prevalence is a fundamental health policy issue. It has been widely recognized that more research is needed to assess the burden of climate change on respiratory diseases that may be translated into public health policies. Previous studies about climate and respiratory symptoms often focus on extreme, short-term climatic conditions [7, 8]. Less is known about the effect of long-term changes in climate on chronic respiratory symptoms.

Finland is a culturally homogeneous country situated in Northern Europe, where the change in climate is rapid [3–5]. The country is long in the north–south direction with considerable within-country climatic differences. The most southern part of Finland has hemiboreal climate whereas a large area in the north has hemiarctic climate [9]. For these reasons, it is a suitable area to investigate the associations between chronic respiratory symptoms and climatic indices. In the present study, we focused on four symptoms that could be clearly defined according to the current literature. We included the long-term average temperature and precipitation in the subjects’ home municipality in the multivariate models about the risk of these symptoms. Finally, we estimated the effect of the anticipated change in the temperature and precipitation on the risk of the symptoms.

## Material and Methods

### The Target Population

This cross-sectional email survey was originally conducted to estimate the prevalence, risk factors and consequences of chronic cough in elderly subjects [10]. It was directed to the members of the Finnish Pensioners` Federation (26 205 members with an email address, mean age 72.7 years, 63.5% female). The subjects were sent an information letter, invitation, and the questionnaire in April 2021. A reminder message was sent 2 weeks later. The data were collected electronically. A filled questionnaire was considered as an informed consent.

The study follows the World Medical Association's Declaration of Helsinki and was approved by the Ethics Committee of Kuopio University Hospital (289/2015). The Finnish Pensioners` Federation permitted the conduct of this study.

### Questionnaire

All subjects answered 62 questions about age, socioeconomic status, lifestyle, recently experienced symptoms, general health, disorders diagnosed by a doctor, medication, and healthcare use within the past year. Current smoking was defined as current daily smoking. Ever smoking was defined as having smoked at least one year on daily basis ever in lifetime. Allergy was defined as self-reported allergy to food, pollens, or animals. Symptom sum was calculated by summing up the following non-respiratory, non-mental symptoms in the past month: Chest pain on exertion, aching joints, back pain, sciatica (back pain that radiates to the leg), toothache, swollen feet, headache, constipation, other gut problems (flatulence, diarrhea), and urinary problems. The subjects were asked whether there was moisture damage identified in their home during the past 12 months. They were also asked about their current home municipality and region.

### Definitions of the Respiratory Symptoms

Chronic rhinosinusitis was present if there was either nasal blockage or discharge, and either reduction/loss of smell or facial pain/pressure for ≥ 3 months within the past year [11]. Chronic cough was defined as current cough which had lasted > 8 weeks [12, 13]. Wheezing with dyspnea was defined as positive answers to both of the following questions: “In the past year (12 months), have you experienced wheezing or a whistling sound when you breathe “ and “Have you experienced a shortness of breath at the same time when your breathing is wheezy or whistling” [14]. Sleep apnea was defined as presence of ≥ 2 of the following features: Loud snoring, daytime tiredness, observed apneas and arterial hypertension (the STOP questionnaire) [15].

### Information About the Home Municipality and the Home Region

The mainland Finland consists of 18 regions, which are divided to municipalities, 293 altogether. For each home region and municipality, the mean annual precipitation, mean winter (November—April) precipitation, mean summer (May—October) precipitation, mean annual temperature, mean winter temperature, and mean summer temperature for the years 2000–2021 were kindly provided by the Finnish Meteorological Institute. In addition, each municipality was classified as urban, semi-urban, or rural, according to the classification by the Statistics Finland [16].

### Statistical Analysis

The relative prevalence of the respiratory symptoms in each region was calculated as the prevalence in the region divided by the mean prevalence in Finland. Two small regions (Ostrobothnia and Central Ostrobothnia) were combined to achieve at least 100 responders for each region.

The associations of the respiratory symptoms with the climatic indices in the home municipality were assessed utilizing bivariate and multivariate logistic regression analyses with a backward directed stepwise procedure. The annual precipitation was expressed as hundreds of mm. In the multivariate models, the following covariates were included: Age, body mass index, gender, allergy, ever smoking daily at least one year, family incomes, the classification of the municipality, and symptom sum. As a sensitivity analysis, the multivariate analyses were also performed separately within nonsmokers and ever smokers.

SPSS software version 29.0.1.0 (IBM SPSS Statistics for Windows) was utilized and a *p* value < 0.05 was considered statistically significant. The descriptive data are expressed as percentages or means and 95% confidence intervals (CI) and the risks as odds ratios (OR) and 95% CIs.

## Results

The response rate was 23.6% (*n* = 6189, Table 1) representing all regions and 282 municipalities. The proportion of missing values was < 2.5%, except for the question about family income (2.9%) and the sleep apnea-related questions (3.1–3.7%). The home municipality and region could not be assessed for 34 subjects (0.5%). 88 subjects (1.4%) reported about moisture damage in their home during the past 12 months.

**Table 1:** The basic characteristics of the 6189 responders

Age (Years)	72.2 (72.08–72.35)
Female gender (%)	66.5
Body mass index (kg/m^2^)	27.4 (27.3–27.5)
Yearly family incomes	
less than 15 000 e (%)	8.2
15 000–40 000 e (%)	53.3
40 000–70 000 e (%)	31.1
70 000–120 000 e (%)	6.7
More than 120 000 e (%)	0.7
Type of current home municipality	
Urban (%)	49.3
Semi-urban (%)	23.8
Rural (%)	26.9
Current smokers (%)	1.8
Ever smokers (%)	36.2
Symptoms of chronic rhinosinusitis (%)	9.7
Current chronic cough (%)	13.7
Wheezing and dyspnea during the last 12 months (%)	8.9
Symptoms of sleep apnea (%)	32.8
Current home region (the numbers on the maps)	
Uusimaa (1) (%)	12.4
Southwest Finland (2) (%)	14.7
Satakunta (4) (%)	2.8
Kanta-Häme (5) (%)	5.1
Pirkanmaa (6) (%)	5.8
Päijät-Häme (7) (%)	2.9
Kymenlaakso (8) (%)	3.4
South Karelia (9) (%)	2.6
South Savo (10) (%)	5.5
North Savo (11) (%)	7.6
North Karelia (12) (%)	4.7
Central Finland (13) (%)	5.9
South Ostrobothnia (14) (%)	8.8
Ostrobothnia and Central Ostrobothnia joined (16) (%)	3.0
North Ostrobothnia (17) (%)	7.9
Kainuu (18) (%)	2.7
Lapland (19) (%)	3.9

The figures are means and 95% confidence intervals or percentages. The numbering of the regions corresponds to the official numbering in Finland and those in the maps

**Fig. 1:**
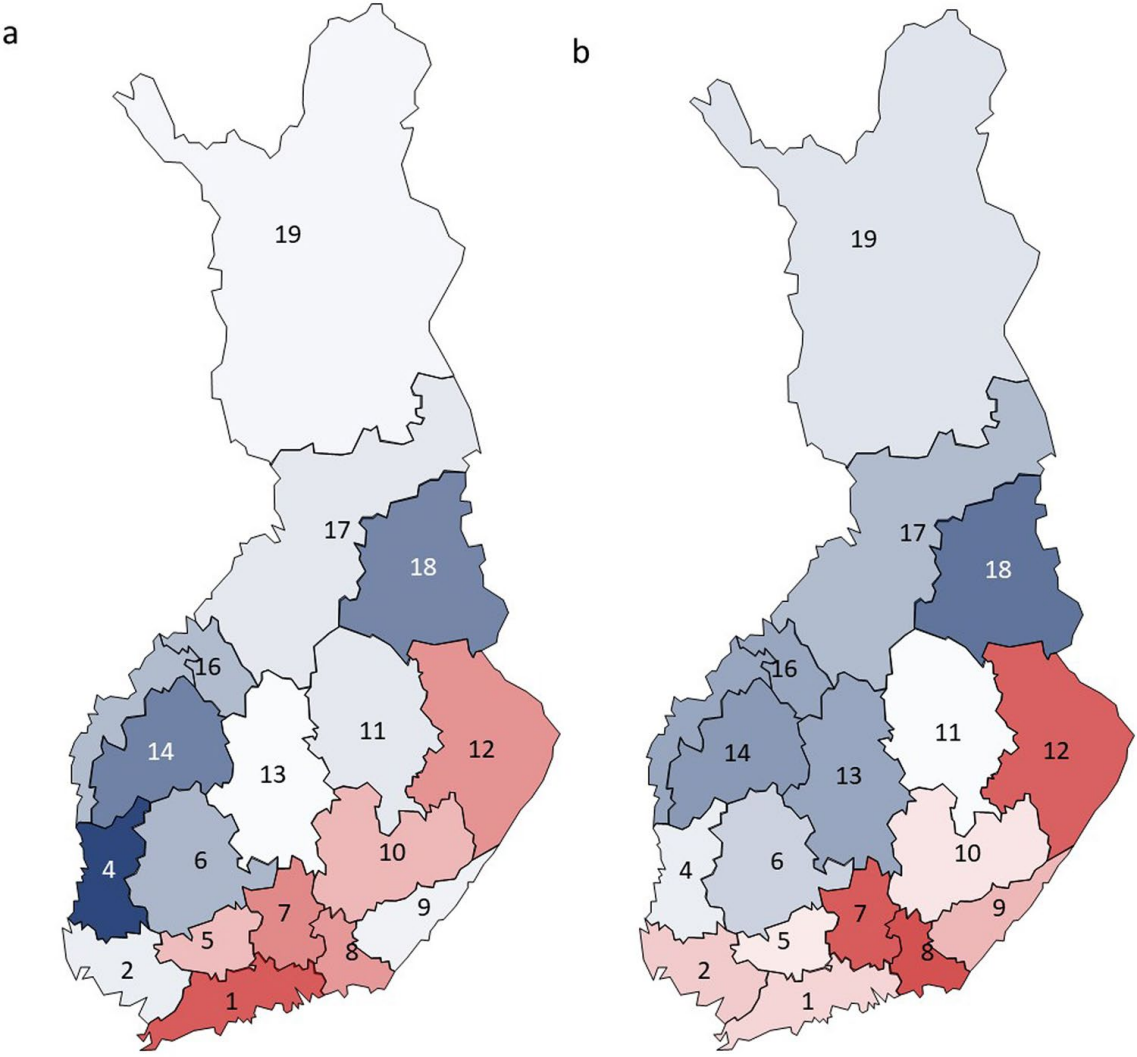
The relative unadjusted prevalence of chronic rhinosinusitis (**a**), and chronic cough (**b**) in the 18 regions of the mainland Finland, according to the current home region. The relative prevalence is calculated as the prevalence in the region divided by the prevalence in whole Finland. In each figure, the red color indicates a prevalence above the mean prevalence in whole Finland, with a darker red color indicating higher prevalence. The blue color indicates a prevalence below the mean prevalence in whole Finland, with a darker blue color indicating lower prevalence. The white color indicates prevalence which is close to the average in whole Finland

**Fig. 2:**
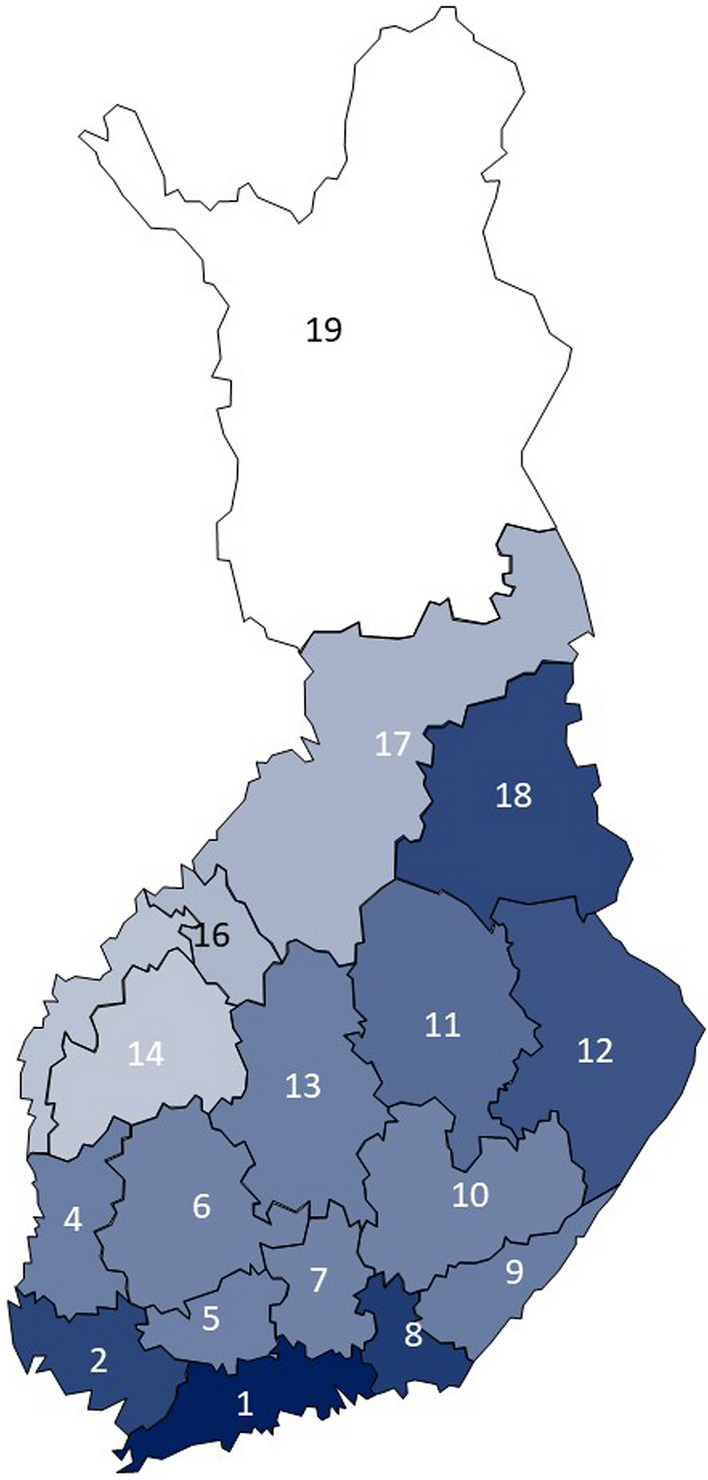
The mean winter precipitations during the years 2000–2021 in the 18 regions of the mainland Finland. The darkest blue color indicates mean winter precipitation of 291 mm (the region with the largest rainfall, Uusimaa, region number 1) and the white color 209 mm (the region with the smallest rainfall, Lapland, region number 19)

**Fig. 3:**
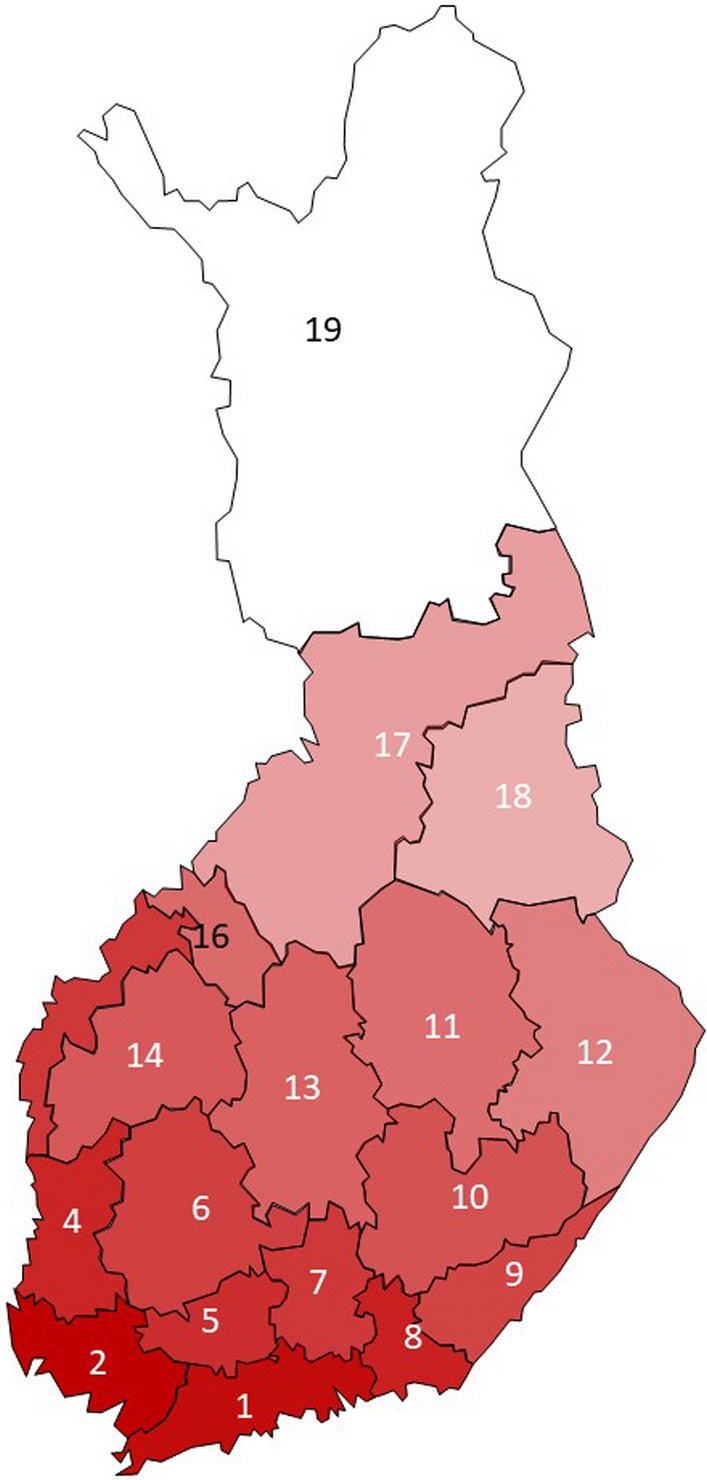
The mean annual temperatures during the years 2000–2021 in the 18 regions of the mainland Finland. The darkest red color indicates mean annual temperature of 6.4 °C (the region with the highest temperature, Varsinais-Suomi, region number 2) and the white color 0.4 °C (the region with the lowest temperature, Lapland, region number 19)

Chronic rhinosinusitis and chronic cough were most prevalent in the south-eastern regions (Fig. 1 a, b). Wheezing with dyspnea and sleep apnea showed no clear regional patterns (data not shown). The long-term winter precipitation was highest in the south-eastern regions (Fig. 2) whereas the long-term temperatures were highest in the south-western regions (Fig. 3).

In the bivariate and multivariate logistic regression analyses, chronic rhinosinusitis and chronic cough were statistically significantly associated with the mean annual precipitation in the home municipality and especially, with the mean winter precipitation (Tables 2, 3). In addition, chronic cough showed a weak association with summer temperature. Wheezing with dyspnea and sleep apnea were not associated with the climatic indices within the total population.

**Table 2:** The bivariate associations between the respiratory symptoms and the mean climatic conditions during the years 2000–2021 in the current home municipality

Climatic indices	Chronic rhinosinusitis	Chronic cough	Wheezing with dyspnea	Sleep apnea
Yearly precipitation	1.58 (1.26–1.98)***	1.34 (1.10 -1.62)**	1.15 (0.91–1.45)	0.98 (0.85–1.13)
Winter precipitation	1.93 (1.40–2.65)***	1.63 (1.24–2.14)***	1.13 (0.81–1.56)	0.98 (0.80–1.20)
Summer precipitation	1.64 (1.07–2.53)*	1.20 (0.83–1.74)	1.35 (0.86–2.12)	0.96 (0.72–1.27)
Yearly temperature	1.03 (0.97–1.10)	1.06 (1.00–1.12)	0.98 (0.91–1.04)	0.99 (0.95–1.03)
Winter temperature	1.02 (0.97–1.07)	1.04 (0.99–1.08)	0.97 (0.93–1.02)	0.99 (0.96–1.03)
Summer temperature	1.08 (0.98–1.18)	1.10 (1.02–1.20)*	0.99 (0.90–1.09)	0.98 (0.93–1.04)

The figures represent odds ratios calculated by logistic regression analyses

The ORs for the precipitation are calculated per 100 mm, the ORs for the temperatures per one ^o^C

*p < 0.05; **p < 0.01; ***p < 0.001

**Table 3:** The multivariate associations between the respiratory symptoms and the mean climatic conditions during the years 2000–2021 in the current home municipality

Climatic indices	Chronic rhinosinusitis	Chronic cough	Wheezing with dyspnea	Sleep apnea
Yearly precipitation	1.55 (1.23–1.96)***	1.33 (1.09 -1.61)**	1.15 (0.90–1.47)	1.00 (0.86–1.17)
Winter precipitation	1.80 (1.30–2.51)***	1.57 (1.19–2.07)**	1.08 (0.76–1.55)	0.97 (0.78–1.21)
Summer precipitation	1.75 (1.12–2.74)*	1.25 (0.86–1.83)	1.46 (0.90–2.37)	1.05 (0.77–1.43)
Yearly temperature	1.02 (0.96–1.09)	1.04 (0.98–1.10)	0.96 (0.90–1.04)	0.97 (0.93–1.02)
Winter temperature	1.01 (0.96–1.06)	1.02 (0.98–1.07)	0.96 (0.91–1.02)	0.98 (0.95–1.02)
Summer temperature	1.06 (0.97–1.17)	1.09 (1.00–1.18)*	0.98 (0.89–1.08)	0.96 (0.90–1.02)

The following covariates were included: Age, body mass index, gender, allergy, ever smoking daily at least one year, family incomes, the classification of the municipality, and symptom sum. The figures represent adjusted odds ratios and 95% confidence intervals calculated by logistic regression analyses

The ORs for the precipitations are calculated per 100 mm, the ORs for the temperatures per one ^o^C

*p < 0.05; **p < 0.01; ***p < 0.001

When the multivariate analyses were performed separately to never smokers and ever smokers (Supplementary file, Tables S1 and S2), the associations of chronic rhinosinusitis and chronic cough with precipitation remained similar. In addition, wheezing with dyspnea and sleep apnea showed weak, but statistically significant negative associations with temperature among ever smokers.

## Discussion

In this study among elderly Finnish subjects, chronic rhinosinusitis and chronic cough were associated with the long-term mean winter precipitation in the home municipality, whereas wheezing with dyspnea and sleep apnea were not associated with any climatic indices. The results suggest different contributions of climate on different chronic respiratory symptoms.

Previous studies about climate and respiratory symptoms often focus on extreme, short-term climatic conditions [8]. A large survey in 32 countries showed that in humid, subtropical regions, anomalously wet conditions increase the risk of cough [17]. A Japanese study found the mean vapor pressure to be the most important environmental factor associated with acute exacerbation of nonallergic rhinitis [18]. Our findings suggest that even mild, but long-lasting or repetitive high precipitation may increase the risks of chronic rhinosinusitis and chronic cough.

In Nordic countries like Finland, much of the winter precipitation comes in the form of snow. Thus, a large proportion of water is in a solid state until spring, when it melts during a relative short period in March–April. At that stage, a large amount of water is released. This may be regarded as minor flooding, though it is a normal annual phenomenon. These minor annual floods may be, at least partly, responsible for the associations of chronic rhinosinusitis and chronic cough with high winter precipitation. Of note, the present survey was performed in April, in the middle of the melting period. This may have affected the results.

The association of high winter precipitation with the risks of chronic rhinosinusitis and chronic cough may mediate via an increase in the indoors humidity. People spend most of their time indoors and indoors absolute humidity correlates well with the outdoors absolute humidity throughout the year [19, 20]. High indoors humidity is well-known to associate with cough and nasal symptoms. Under such conditions, the building occupants may be exposed to increased levels of microbial agents such as fungal spores, formaldehyde from building materials, as well as odors, microparticles and vapors [21–24]. However, just 1.4% of the subjects in the present survey reported about moisture damage in their home. Due to the low prevalence of moisture damage, its regional distribution could not be assessed in the present study.

Another explanation for the association between high precipitation and respiratory symptoms is that high precipitation may increase the pollen concentrations. The association between precipitation and the pollen load is complex. Rainfall during the flowering season decreases the pollen load whereas high precipitation during months preceding the pollination may increase it [25]. Winter precipitation probably precedes pollination and therefore, high winter precipitation could intensify the pollination. Furthermore, thunderstorms have been associated with increasing allergic asthma outbreaks mediated through suddenly increased pollen and fungal spore exposure [7].

It may be plausible to assume that the direct or indirect effects of excessive ambient moisture on respiratory symptoms mediate by altering the function of the airway epithelium. Chronic rhinosinusitis, chronic cough, and wheezing with dyspnea all may involve a dysfunctional airway epithelial barrier [26]. Therefore, it is interesting that only chronic rhinosinusitis and chronic cough were associated with high winter precipitation. We suggest three possible explanations. Firstly, the nose plays a primary role within the airways, working as a filter and air-conditioner [27]. Thus, it is probable that the effects of high indoors humidity or high pollen load affects more the nose than the lower airways [28]. Secondly, chronic rhinosinusitis is a well-known cause for chronic cough [12, 13]. Thirdly, wheezing with dyspnea is typical for asthma, which is a very strongly genetically determined disorder [29, 30]. Thus, long-term environmental factors may play a relatively minor role in asthma than in chronic rhinosinusitis and chronic cough though they are important factors affecting asthma development and exacerbations.

The recently updated climate change scenarios indicate that the winter precipitation may increase up to 40% during the current century in Finland, indicating almost 100 mm increase [4]. Our multivariate model suggest that this could increase the risk of chronic rhinosinusitis by 80% and the risk of chronic cough by 57%. These are alarming figures given the high prevalence and the high health resource utilization due to these symptoms [6]. However, projections to the future are complicated and our results should be interpreted with caution. For example, despite the increasing winter precipitation, the snow cover tends to get thinner in the Northern Hemisphere due to warming of the winters [31]. This probably decreases the amounts of water released during the melting period in spring. In addition, climate change probably leads to many other environmental phenomena. These include the intensification of pollen and other bioallergenic proteins production, including bacteria, viruses, animal dander, insects, molds, and plant species. Furthermore, climate change may also influence the ambient concentrations of air pollutants including carbon monoxide, lead, nitrogen dioxide, ozone, particulate matter, and sulfur dioxide. These variables could not be taken into account in the present study but are a capable to affect, and probably increase further, the risks of chronic rhinosinusitis and chronic cough in the future [8].

In the present study, the associations between long-term temperatures and respiratory symptoms were weak or absent. This may be since people spend most of their time indoors and that in cool climates like that in Finland, indoors temperatures are kept rather stable throughout the year. Among ever smokers, wheezing with dyspnea and sleep apnea showed weak associations with cold ambient temperature. Cold air is a well-known trigger of bronchoconstriction [28] and symptoms of sleep apnea have been documented to increase during the cool periods of the year [32, 33]. It remains obscure why these associations were not present in the never smokers of the present study.

This cross-sectional survey can only reveal associations, not causal relationships. We did not have information about local pollen counts for the present study, which can be regarded as a weakness. Other weaknesses of the present study include the limited generalizability of the results: They may only apply to countries with cool climate and an increase in precipitation due to climate change. In many other areas of the word, the climate change probably decreases precipitation [1]. Furthermore, the present population was old, and the results may not be directly applicable to younger subjects. The participation rate was low, but the age and gender distribution of the responders closely resembled that of the target population.

The main strength of the present study is the accurate information about the long-term mean temperatures and precipitations in each subjects’ home municipality. The respiratory symptoms were strictly defined as currently suggested in the literature. Furthermore, many factors that could affect the risk of respiratory symptoms could be considered in the multivariate analyses: Allergy, smoking, socio-economical state, urban versus rural municipality, and the individual differences in experiencing and reporting of bodily sensations, which was covered by the variable ‘symptom sum’ [34, 35].

In conclusion, chronic rhinosinusitis and chronic cough showed strong associations with long-term winter precipitation in the home municipality. Given the anticipated rapid increase in winter precipitation in Northern America and Northern Europe due to climate change, these symptoms may become more prevalent and present an increasing burden on primary health care. However, more reliable projections for the future would require more comprehensive information about other climate change-associated phenomena.

## Supplementary Information

Below is the link to the electronic supplementary material.

Supplementary file1 (DOCX 47 KB)

## References

[1] Lehtonen I, Jylhä K (2019) Tendency towards a more extreme precipitation climate in the Coupled Model Intercomparison Project Phase 5 models. Atmospheric Sci Lett. 10.1002/asl.895

[2] Çelebi Sözener Z, Treffeisen ER, Özdel Öztürk B, Schneider LC (2023) Global warming and implications for epithelial barrier disruption and respiratory and dermatologic allergic diseases. J Allergy Clin Immunol 152:1033–1046. 10.1016/j.jaci.2023.09.001 37689250 10.1016/j.jaci.2023.09.001 PMC10864040

[3] McCrystall MR, Stroeve J, Serreze M et al (2021) New climate models reveal faster and larger increases in Arctic precipitation than previously projected. Nat Commun 12:6765. 10.1038/s41467-021-27031-y 34848697 10.1038/s41467-021-27031-y PMC8633026

[4] Ruosteenoja K, Jylha K (2021) Projected climate change in Finland during the 21st century calculated from CMIP6 model simulations. Geophysica 56:39–69

[5] Kirchmeier-Young MC, Zhang X (2020) Human influence has intensified extreme precipitation in North America. Proc Natl Acad Sci U S A 117:13308–13313. 10.1073/pnas.1921628117 32482861 10.1073/pnas.1921628117 PMC7306817

[6] Finley CR, Chan DS, Garrison S et al (2018) What are the most common conditions in primary care? Systematic review. Can Fam Physician Med Fam Can 64:832–840 PMC6234945 30429181

[7] Vicedo-Cabrera AM, Melén E, Forastiere F et al (2023) Climate change and respiratory health: a European Respiratory Society position statement. Eur Respir J 62:2201960. 10.1183/13993003.01960-2022 37661094 10.1183/13993003.01960-2022

[8] Balakrishnan B, Callahan SJ, Cherian SV et al (2023) Climate change for the pulmonologist: a focused review. Chest 164:963–974. 10.1016/j.chest.2023.04.009 37054776 10.1016/j.chest.2023.04.009

[9] Finnish Meteorological Institute Suomen ilmastovyöhykkeet - Ilmatieteen laitos. https://www.ilmatieteenlaitos.fi/suomen-ilmastovyohykkeet. Accessed 7 Nov 2023

[10] Kaulamo JT, Lätti AM, Koskela HO (2022) Cough in the elderly during the COVID-19 pandemic. Lung 200:161–168. 10.1007/s00408-022-00525-2 35298689 10.1007/s00408-022-00525-2 PMC8927524

[11] Fokkens WJ, Lund VJ, Mullol J et al (2012) European position paper on rhinosinusitis and nasal polyps 2012. RhinologySupplement 50:1–298

[12] Irwin RS, French CL, Chang AB, Altman KW (2018) Classification of cough as a symptom in adults and management algorithms: CHEST guideline and expert panel report. Chest 153:196–209 29080708 10.1016/j.chest.2017.10.016 PMC6689094

[13] Morice AH, Millqvist E, Bieksiene K et al (2020) ERS guidelines on the diagnosis and treatment of chronic cough in adults and children. Eur Respir J 55:1901136. 10.1183/13993003.01136-2019 31515408 10.1183/13993003.01136-2019 PMC6942543

[14] Sa-Sousa A, Jacinto T, Azevedo LF et al (2014) Operational definitions of asthma in recent epidemiological studies are inconsistent. Clin Transl Allergy 4:24–24. 10.1186/2045-7022-4-24 25136441 10.1186/2045-7022-4-24 PMC4136946

[15] Chung F, Yegneswaran B, Liao P et al (2008) STOP questionnaire: a tool to screen patients for obstructive sleep apnea. Anesthesiology 108:812–821. 10.1097/ALN.0b013e31816d83e4 18431116 10.1097/ALN.0b013e31816d83e4

[16] Statistics Finland Classifications. https://www.stat.fi/en/luokitukset/. Accessed 7 Nov 2023

[17] Dimitrova A, McElroy S, Levy M et al (2022) Precipitation variability and risk of infectious disease in children under 5 years for 32 countries: a global analysis using Demographic and Health Survey data. Lancet Planet Health 6:e147–e155. 10.1016/S2542-5196(21)00325-9 35150623 10.1016/S2542-5196(21)00325-9

[18] Hoshino T, Hoshino A, Nishino J (2015) Relationship between environment factors and the number of outpatient visits at a clinic for nonallergic rhinitis in Japan, extracted from electronic medical records. Eur J Med Res 20:60. 10.1186/s40001-015-0151-3 26152217 10.1186/s40001-015-0151-3 PMC4502595

[19] Nguyen JL, Schwartz J, Dockery DW (2014) The relationship between indoor and outdoor temperature, apparent temperature, relative humidity, and absolute humidity. Indoor Air 24:103–112. 10.1111/ina.12052 23710826 10.1111/ina.12052 PMC3791146

[20] Nguyen JL, Dockery DW (2016) Daily indoor-to-outdoor temperature and humidity relationships: a sample across seasons and diverse climatic regions. Int J Biometeorol 60:221–229. 10.1007/s00484-015-1019-5 26054827 10.1007/s00484-015-1019-5 PMC4674394

[21] Park J-H, Cox-Ganser JM (2011) Mold exposure and respiratory health in damp indoor environments. Front Biosci Elite Ed 3:757–771. 10.2741/e284 21196349 10.2741/e284

[22] Mendell MJ, Mirer AG, Cheung K et al (2011) Respiratory and allergic health effects of dampness, mold, and dampness-related agents: a review of the epidemiologic evidence. Environ Health Perspect 119:748–756. 10.1289/ehp.1002410 21269928 10.1289/ehp.1002410 PMC3114807

[23] Jaakkola MS, Quansah R, Hugg TT et al (2013) Association of indoor dampness and molds with rhinitis risk: a systematic review and meta-analysis. J Allergy Clin Immunol 132:1099-1110.e18. 10.1016/j.jaci.2013.07.028 24028857 10.1016/j.jaci.2013.07.028

[24] Guarnieri G, Olivieri B, Senna G, Vianello A (2023) Relative humidity and its impact on the immune system and infections. Int J Mol Sci 24:9456. 10.3390/ijms24119456 37298409 10.3390/ijms24119456 PMC10253274

[25] Mousavi F, Oteros J, Shahali Y, Carinanos P (2024) Impacts of climate change on allergenic pollen production: a systematic review and meta-analysis. Agric For Meteorol. 10.1016/j.agrformet.2024.109948

[26] Hellings PW, Steelant B (2020) Epithelial barriers in allergy and asthma. J Allergy Clin Immunol 145:1499–1509. 10.1016/j.jaci.2020.04.010 32507228 10.1016/j.jaci.2020.04.010 PMC7270816

[27] Marseglia GL, Merli P, Caimmi D et al (2011) Nasal disease and asthma. Int J Immunopathol Pharmacol 24:7–12. 10.1177/03946320110240S402 22032779 10.1177/03946320110240S402

[28] Koskela HO (2007) Cold air-provoked respiratory symptoms: the mechanisms and management. Int J Circumpolar Health 66:91–100 17515249 10.3402/ijch.v66i2.18237

[29] El-Husseini ZW, Gosens R, Dekker F, Koppelman GH (2020) The genetics of asthma and the promise of genomics-guided drug target discovery. Lancet Respir Med 8:1045–1056. 10.1016/S2213-2600(20)30363-5 32910899 10.1016/S2213-2600(20)30363-5

[30] Skadhauge LR, Christensen K, Kyvik KO, Sigsgaard T (1999) Genetic and environmental influence on asthma: a population-based study of 11,688 Danish twin pairs. Eur Respir J 13:8–14. 10.1183/09031936.99.13100899 10836316 10.1183/09031936.99.13100899

[31] Gottlieb AR, Mankin JS (2024) Evidence of human influence on Northern Hemisphere snow loss. Nature 625:293–300. 10.1038/s41586-023-06794-y 38200299 10.1038/s41586-023-06794-y PMC10781623

[32] Ingram DG, Matthews CK, Plante DT (2015) Seasonal trends in sleep-disordered breathing: evidence from Internet search engine query data. Sleep Breath Schlaf Atm 19:79–84. 10.1007/s11325-014-0965-1 10.1007/s11325-014-0965-1 24595717

[33] Liu W-T, Wang Y-H, Chang L-T et al (2022) The impacts of ambient relative humidity and temperature on supine position-related obstructive sleep apnea in adults. Environ Sci Pollut Res Int 29:50755–50764. 10.1007/s11356-022-18922-8 35239114 10.1007/s11356-022-18922-8

[34] Barsky AJ, Silbersweig DA (2023) The amplification of symptoms in the medically Ill. J Gen Intern Med 38:195–202. 10.1007/s11606-022-07699-8 35829874 10.1007/s11606-022-07699-8 PMC9849656

[35] Creed F (2022) The predictors of somatic symptoms in a population sample: the lifelines cohort study. Psychosom Med 84:1056–1066. 10.1097/PSY.0000000000001101 35797562 10.1097/PSY.0000000000001101

