# Supplementary material for: The Associations of Long-Term Temperature and Precipitation with Chronic Respiratory Symptoms: Projections for the Changing Climate
Source: Lung. 2024 Nov 29;203(1):7. doi: 10.1007/s00408-024-00763-6 (PMC11607035; doi:10.1007/s00408-024-00763-6)
Supplement: Supplementary file 1 — Supplementary file1 (DOCX 47 KB) [file 408_2024_763_MOESM1_ESM.docx]

SUPPLEMENTARY FILE

Table S1. The multivariate associations between the respiratory symptoms and the mean climatic conditions during the years 2000-2021 in the current home municipality in 3906 subjects who had never smoked on daily basis. The following covariates were included: Age, body mass index, gender, allergy, family incomes, the classification of the municipality, and symptom sum. The figures represent adjusted odds ratios and 95 % confidence intervals calculated by logistic regression analyses.

| Climatic indices | Chronic  rhinosinusitis | Chronic cough | Wheezing with dyspnea | Sleep apnea |
| --- | --- | --- | --- | --- |
| Yearly precipitation | 1.59 (1.16-2.17)** | 1.31 (1.01-1.69)* | 1.18 (0.85-1.65) | 0.97 (0.79-1.19) |
| Winter precipitation | 1.80 (1.17-2.79 ** | 1.51 (1.05-2.18)* | 1.29 (0.80-2.09) | 1.05 (0.79-1.41) |
| Summer precipitation | 1.87 (1.03-3.41)* | 1.25 (0.75-2.07) | 1.17 (0.61-2.23) | 0.79 (0.53-1.18) |
| Yearly temperature | 1.03 (0.94-1.12) | 1.03 (0.95-1.11) | 1.02 (0.92-1.13) | 1.01 (0.95-1.08) |
| Winter temperature | 1.02 (0.95-1.09) | 1.01 (0.95-1.07) | 1.00 (0.93-1.08) | 1.01 (0.97-1.06) |
| Summer temperature | 1.07 (0.94-1.21) | 1.10 (0.98-1.22) | 1.07 (0.93-1.23) | 1.00 (0.92-1.09) |

The ORs for the precipitations are calculated per 100 mm, the ORs for the temperatures per one ^o^C.

* p < 0.05; ** p < 0.01

Table S2. The multivariate associations between the respiratory symptoms and the mean climatic conditions during the years 2000-2021 in the current home municipality among 2217 subjects, who had smoked at least one year on daily basis ever in lifetime. The following covariates were included: Age, body mass index, gender, allergy, family incomes, the classification of the municipality, and symptom sum. The figures represent adjusted odds ratios and 95 % confidence intervals calculated by logistic regression analyses.

| Climatic indices | Chronic  rhinosinusitis | Chronic cough | Wheezing with dyspnea | Sleep apnea |
| --- | --- | --- | --- | --- |
| Yearly precipitation | 1.48 (1.04-2.10)* | 1.39 (1.03 -1.87)* | 1.11 (0.76-1.60) | 1.06 (0.83-1.35) |
| Winter precipitation | 1.76 (1.07-2.90)* | 1.74 (1.13-2.67)* | 0.87 (0.51-1.47) | 0.89 (0.63-1.27) |
| Summer precipitation | 1.55 (0.79-3.03) | 1.35 (0.75-2.44) | 1.87 (0.91-3.82) | 1.51 (0.95-2.41) |
| Yearly temperature | 1.01 (0.91-1.12) | 1.06 (0.97-1.16) | 0.91 (0.82-1.00) | 0.92 (0.86-0.99)* |
| Winter temperature | 1.00 (0.92-1.08) | 1.04 (0.98-1.12) | 0.92 (0.85-1.00)* | 0.94 (0.89-0.99)* |
| Summer temperature | 1.05 (0.91-1.21) | 1.09 (0.97-1.23) | 0.90 (0.78-1.03) | 0.90 (0.82-0.99)* |

The ORs for the precipitations are calculated per 100 mm, the ORs for the temperatures per one ^o^C.

* p < 0.05
